# The Diverse Iron Distribution in Eudicotyledoneae Seeds: From Arabidopsis to Quinoa

**DOI:** 10.3389/fpls.2018.01985

**Published:** 2019-01-15

**Authors:** Miguel Angel Ibeas, Susana Grant-Grant, Maria Fernanda Coronas, Joaquín Ignacio Vargas-Pérez, Nathalia Navarro, Isidro Abreu, Hiram Castillo-Michel, Natalia Avalos-Cembrano, Julio Paez Valencia, Fernanda Perez, Manuel González-Guerrero, Hannetz Roschzttardtz

**Affiliations:** ^1^Facultad de Ciencias Biológicas, Pontificia Universidad Católica de Chile, Santiago, Chile; ^2^Centro de Biotecnología y Genómica de Plantas (UPM-INIA), Universidad Politécnica de Madrid, Madrid, Spain; ^3^ID21, European Synchrotron Radiation Facility (ESRF), Grenoble, France; ^4^Department of Botany, University of Wisconsin–Madison, Madison, WI, United States; ^5^Departamento de Ecología, Pontificia Universidad Católica de Chile, Santiago, Chile

**Keywords:** apomorphy, Arabidopsis, quinoa, embryo, iron, phylogeny

## Abstract

Seeds accumulate iron during embryo maturation stages of embryogenesis. Using *Arabidopsis thaliana* as model plant, it has been described that mature embryos accumulate iron within a specific cell layer, the endodermis. This distribution pattern was conserved in most of the analyzed members from Brassicales, with the exception of the basal *Vasconcellea pubescens* that also showed elevated amounts of iron in cortex cells. To determine whether the *V. pubescens* iron distribution was indicative of a wider pattern in non-Brassicales Eudicotyledoneae, we studied iron distribution pattern in different embryos belonging to plant species from different Orders from Eudicotyledoneae and one basal from Magnoliidae. The results obtained indicate that iron distribution in *A. thaliana* embryo is an extreme case of apomorphic character found in Brassicales, not-extensive to the rest of Eudicotyledoneae.

## Introduction

Increased iron content in seeds is an important agronomic trait. This is due to the relevance of this element in seed production (Marschner, 2005; Roschzttardtz et al., 2011), embryo development, and seedling germination and growth (Lanquar et al., 2005; Kim et al., 2006), as well as in human nutrition (Murgia et al., 2012). In spite of this essential role, the prevalent low iron bioavailability in the soil of most of the main agricultural areas of the world, limits plant productivity, fertility, and even germination rates (Guerinot and Yi, 1994). Consequently, a substantial effort has been dedicated to unraveling the molecular bases controlling iron homeostasis, how plants incorporate and distribute iron throughout their organs, and how iron is stored in the seeds (Walker and Waters, 2011; Kobayashi and Nishizawa, 2012; Grillet et al., 2014; Flis et al., 2016; Tsai and Schmidt, 2017).

Iron distribution in seeds has been studied in monocots and Eudicotyledoneae. Using rice and wheat as models it was concluded that most of the iron in monocot plants is stored in the aleurone layer (Iwai et al., 2012; De Brier et al., 2016). In Eudicotyledoneae, the majority of work has been carried out in Arabidopsis. It has been estimated that approximately 50% of the seed iron content is stored in endodermal cells (Roschzttardtz et al., 2009; Schnell Ramos et al., 2013). Results from experiments using multiple iron-imaging methods showed that the vast majority of the seed embryo iron is located in vacuoles (Kim et al., 2006; Roschzttardtz et al., 2009; Schnell Ramos et al., 2013). This is in contrast to other plant tissues in which iron has also been detected in plastids, associated with ferritin, and in nucleoli (Roschzttardtz et al., 2011, 2013).

Iron accumulates steadily in the endodermal vacuole during the maturation stage of embryo development, and is subsequently used during post-germinative growth (Lanquar et al., 2005; Roschzttardtz et al., 2009). The defects shown by the *vit1* and *nramp3 nramp4* mutants under iron deficiency conditions suggest that proper iron storage seems essential for seed germination and post-germinative growth (Lanquar et al., 2005; Kim et al., 2006; Mary et al., 2015). These mutant plants are defective in iron loading into the vacuoles of endodermal cells during embryo maturation, a process mediated by the transporter AtVIT1 (VACUOLAR IRON TRANSPORTER1) (Kim et al., 2006); or in iron recovery from vacuoles in these cells via the AtNRAMP3 and AtNRAMP4 (NATURAL RESISTANCE ASSOCIATED MACROPHAGE PROTEIN3 and 4) transporters during germination (Lanquar et al., 2005).

Closely related Brassicaceae embryos accumulate iron very similarly to Arabidopsis, i.e., in vacuoles of cells surrounding the embryo provasculature (Ibeas et al., 2017). However, reports on another Eudicotyledoneae, the legume *Phaseolus vulgaris*, showed broader iron localization. While iron hotspots were detected in cotyledons in a pattern resembling the provasculature, the metal was also evenly distributed in the rest of the organ (Cvitanich et al., 2010). This alternative way of accumulating iron in the seed could indicate that the storage of this nutrient in Eudicotyledoneae is more diverse than what has been reported to date. Considering the importance of Eudicotyledoneae as crops, and the need to improve the iron content in the edible parts (DeFries et al., 2015), we set to study iron distribution in embryos from a wide range of orders belonging to dicotyledonous plants. Our results show that the iron distribution pattern in the Arabidopsis embryo is not extended to all Eudicotyledoneae, it rather seems to be a derived character only observed in the Brassicaceae family. We also found that Caryophyllales has a distinct iron distribution pattern and that iron loading during *Chenopodium quinoa* seed development is different than what we had previously observed in Brassicaceae (Ibeas et al., 2017), suggesting there might be different strategies for the storage of iron in these seeds.

## Materials and Methods

### Plant Material and Growth Conditions

Most of the seeds used in this study were purchased at a local market or harvested in public gardens. Some seeds were collected in the field and identified before being deposited in the herbarium at the Departamento de Ecología, Pontificia Universidad Católica de Chile. The list of species used in this study is in Supplementary Table S1. *C. quinoa* plants were grown on soil in a greenhouse at 23°C under long-day condition (16-h/8-h day/night cycle).

### Histochemical Staining of Iron With Perls/DAB Method

Seed embryos from the species indicated in Supplementary Table S1 or *C. quinoa* seeds at different developmental stages were isolated and fixed with 2% *w*/*v* paraformaldehyde in 1 mM phosphate buffer pH 7.0 for 45 min. The following steps were performed according to Roschzttardtz et al. (2009) and Ibeas et al. (2017).

### Synchrotron μ-XRF

Quinoa seeds were embedded in OCT (Optimal Cutting Temperature) resin and plunge-frozen in isopentane chilled in liquid nitrogen. Longitudinal sections (30 μm) were prepared using a cryomicrotome (Leica, RM2265-LN22) at –50°C and immediately imaged at beamline ID21 at the ESRF (European Synchrotron Radiation Facility, Grenoble, France). Elemental distribution was mapped by micro X-ray fluorescence (μXRF) under cryogenic conditions (Cotte et al., 2017). The beam was focused with the use of KB mirrors to a size of 0.5 × 0.9 μm^2^ (V × H). The fluorescence signal was detected using an 80 mm^2^ active area SGX Si drift detector with a Be window. Two photodiodes were used to measure the incident and transmitted beam intensities. Scans were acquired with an energy of 7.2 keV (Fe k-edge) with a dwell time of 100 ms per pixel, and a pixel size of 2 μm × 2 μm. RGB color maps and Fe, Mn, and P elemental maps were created using PyMca software (Soléa et al., 2007).

### Phylogenetic Analysis

A phylogenetic tree of the species in the data set was assembled using the phylogeny of angiosperms at family level, from the Angiosperm Phylogeny Group (The Angiosperm Phylogeny Group et al., 2016). To resolve relationships within Brassicaceae we followed Guo et al. (2017), which uses 77 protein coding regions. Branch lengths were set to 1.0. Species were assigned to four categories according to Fe location in seed embryos: endodermis; inner layers of cortex, external cortex or protodermis. Ancestral states of iron location were reconstructed using Mesquite (Maddison and Maddison, 2007) based on one-parameter model.

## Results

### Differences in Iron Distribution in Embryos of the Order Brassicales

Analyses on species from the Brassicaceae family showed that their embryos have similar subcellular iron distribution in cotyledons and hypocotyl (Ibeas et al., 2017). In *Arabidopsis thaliana*, *Camelina sativa*, *Nasturtium officinale, Lepidium sativum*, and *Brassica napus* dry seed embryos, iron accumulates in the vacuoles of cells surrounding the provasculature. Some differences in the number of cells that store iron were observed in the hypocotyl: while Arabidopsis only presented a one cell layer, the other species have two or more (Roschzttardtz et al., 2009; Ibeas et al., 2017).

**FIGURE 1:**
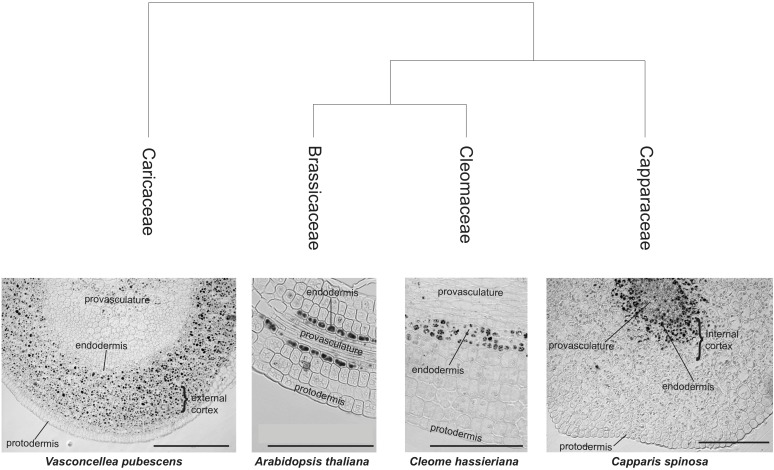
Iron distribution in Brassicales embryos. Iron distribution in hypocotyls of four Brassicales species were mapped on the phylogenetic tree modified from Hall et al. (2004). The analysis of sections using Perls/DAB of Brassicaceae family species was previously described in Ibeas et al. (2017). Iron is accumulated, in addition to endodermis, in the cortex cells (internal cortex, close to endodermis, and close to the protodermis, external cortex). A section of *A. thaliana* hypocotyl stained with Perls/DAB is shown. Bars in panels correspond to 100 μm.

We extended this study to members of other families within the Brassicales order. We performed Perls/DAB staining on *Cleome hassleriana* (Cleomaceae Family) and *Capparis spinosa* (Capparaceae Family). Embryo sections revealed that iron accumulates in cells that surround the provasculature in a similar manner as embryos from Brassicaceae species (Figure 1). Some differences were observed in *C. spinosa*, where a group of cells between provasculature regions in cotyledons showed iron accumulation in vacuoles (Supplementary Figure S1). Surprisingly, when analyzing the member of the Caricaceae family *V. pubescens* (a more ancestral Brassicales), we observed that several cortex cells accumulate iron. This pattern sets it apart from the rest of Brassicales embryos studied to date and suggests that Brassicaceae embryo cortex cells may also accumulate iron (Figure 1).

### Iron Distribution in Embryos From Different Orders of Eudicotyledoneae

**FIGURE 2:**
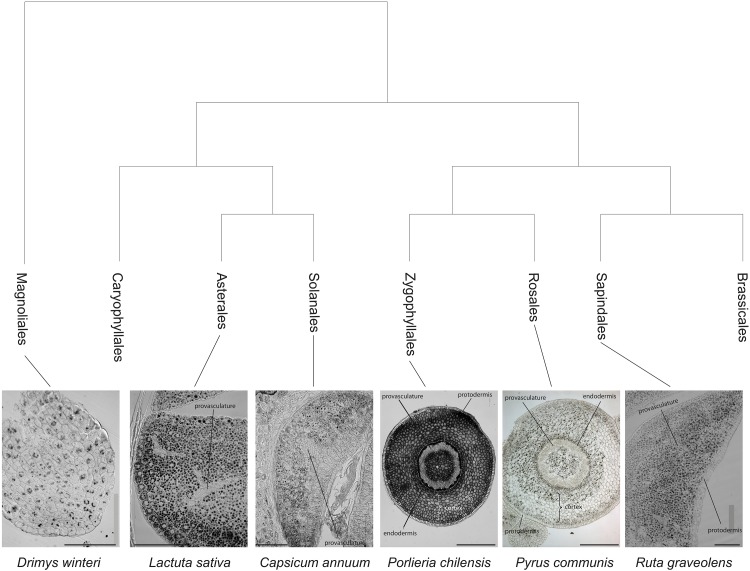
Iron distribution in Eudicotyledoneae mature embryos. Iron distribution was revealed by Perls/DAB staining of hypocotyls (*Porlieria chilensis* and *Pyrus communis*) or cotyledons sections (*Ruta graveolens*, *Capsicum annuum*, and *Lactuca sativa*). The phylogenetic tree was assembled from The Angiosperm Phylogeny Group et al. (2016). Bars in panels correspond to 100 μm.

To inquire whether the observation that *V. pubescencs* has a differential iron distribution is an exception or an ancestral trait lost at some point in the Brassicales order, Perls/DAB analyses were carried out in embryos from six representative Eudicotyledoneae Orders (Sapindales, Rosales, Zygophyllales, Solanales, Asterales, and Caryophyllales) and one basal specie from Magnoliidae (Magnoliales). Interestingly, the analysis of embryos from those orders showed that iron accumulates in several cell layers including endodermis and cortex cells. In some cases, iron was also detected in the protodermis (Solanales, Asterales, and Magnoliales; Figure 2). These results indicate that the Arabidopsis embryo has an uncommon iron distribution compared to embryos of seed plants from other Orders of the Eudicotyledoneae class. Moreover, a more detailed analysis of *Porlieria chilensis* Perls/DAB-stained section shows that iron also seems to accumulate extracellularly, in what might be described as the cortex apoplast (Figure 2).

### Iron Distribution in Caryophyllales Embryos

**FIGURE 3:**
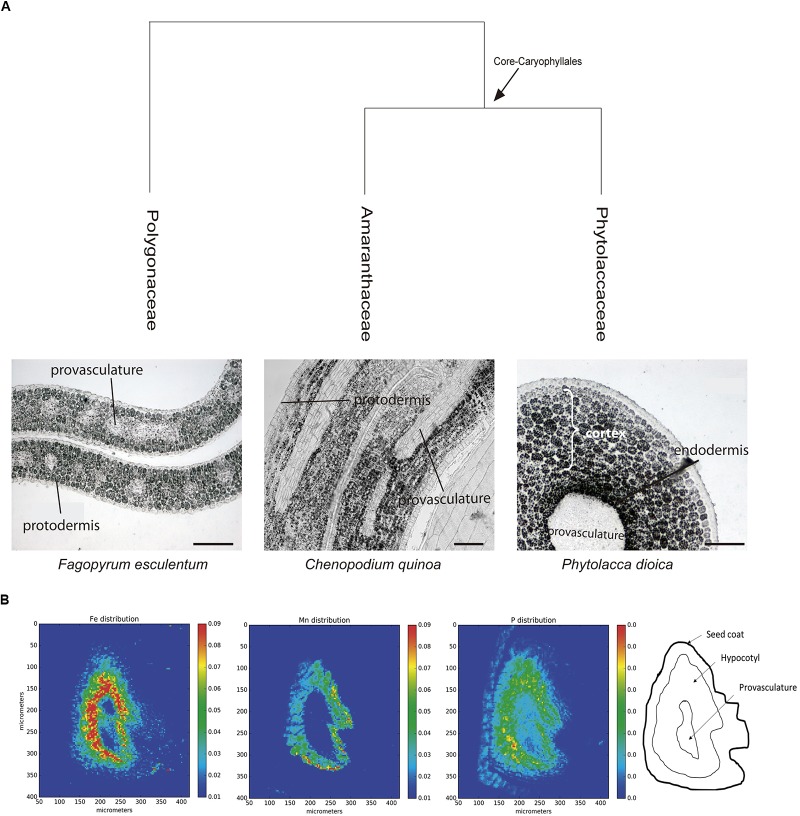
**(A)** Iron distribution in Caryophyllales mature embryos. Perls/DAB stained embryo sections from polygonaceae, amaranthaceae, and phytolaccaceae families (*Fagopyrum esculentum*, *Chenopodium quinoa*, and *Phytolacca dioica*, respectively). Bars in panels correspond to 50 μm. **(B)** Iron detection in hypocotyl Quinoa dry seed section using synchrotron-based micro X-ray fluorescence (SμXRF). P, Mn, and Fe distribution are shown.

In order to evaluate iron distribution in species from other orders, seed embryos of several species belonging to Caryophyllales were used to determine whether iron distribution might differ within the same order. Figure 3A shows embryos from *Fagopyrum esculentum*, *C. quinoa* and *Phytolacca dioica.* In all cases, embryos were isolated and fixed, and then thin sections were analyzed, in particular hypocotyl and cotyledon. Using Perls/DAB staining we found that in all the species analyzed, iron was present in several embryo cell types, including endodermis and cortex cells. This result indicates that this pattern of iron distribution is conserved within this order (Figure 3A). We also analyzed embryos from *Spinacia oleracea, Beta vulgaris*, and *Rheum rhabarbarum* (Supplementary Table S1).

Next, we used synchrotron-based micro X-ray fluorescence (SμXRF) to independently confirm iron distribution using Perls/DAB staining in *C. quinoa* seeds. Dry seeds were sectioned and analyzed by S-μXRF, showing iron accumulation in several cell layers in hypocotyl but excluding provasculature (Figure 3B). S-μXRF also showed Mn accumulation in several cell layers of *C. quinoa* hypocotyl (probably cortex) in contrast with the restricted localization of Mn in the subepidermal layer of plant model *A. thaliana* (Kim et al., 2006; Punshon et al., 2012; Schnell Ramos et al., 2013; Eroglu et al., 2017).

### Iron Distribution During *Chenopodium quinoa* Seed Development

Previously, our group reported for the first time that some Brassicaceae species accumulate iron in two embryo cell types (endodermis and one cell layer of cortex). We also showed that in early stages of *B. napus* seed development, iron accumulates in nuclei of the free cell endosperm and in all embryo cell types. Later, iron is relocated to cytoplasmic structures, and finally, at the mature stage, iron is accumulated in vacuoles of the endodermis and cortex (Ibeas et al., 2017).

Because the iron distribution pattern in Caryophyllales is different from the pattern described above, we used *C. quinoa* as a model to study iron localization during seed development. *C. quinoa* is an emerging crop that has potential health benefits and an exceptional nutritional value. We analyzed four different stages of seed maturation in *C. quinoa*: early stages (between 3 and 7 days postanthesis), an intermediate stage (14 days postanthesis/cotyledon stage) and a late stage (21 days postanthesis/mature stage). In order to describe the seed structures where iron accumulates before being loaded into the embryo, we used whole seeds including seed coat, perisperm and embryo in our analyses.

**FIGURE 4:**
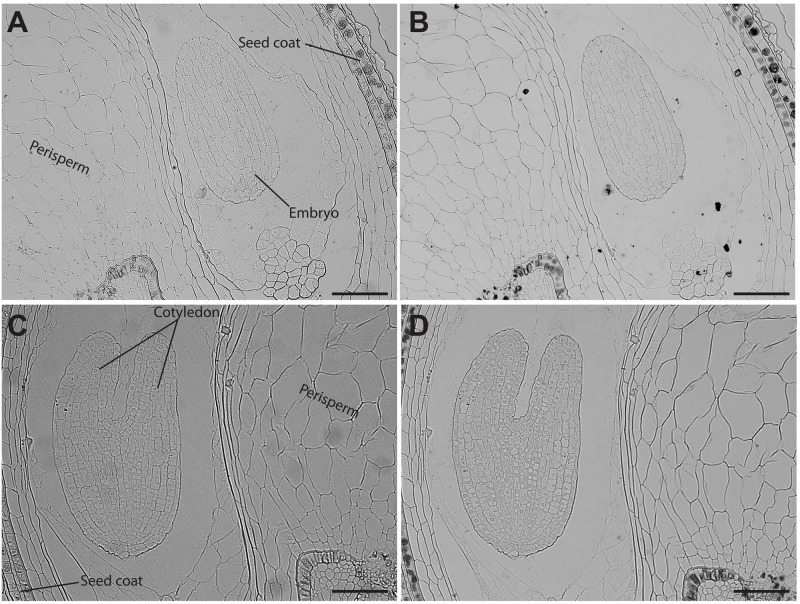
Iron distribution in *C. quinoa* seeds in early developmental stages. *C. quinoa* seeds 3 and 7 days postanthesis were collected from fruits, embedded in Technovit resin, sectioned (3 μm) and then stained with Perls/DAB **(B**,**D)**. Unstained sections were used as control **(A**,**C)**. The scale bar represents 100 μm.

**FIGURE 5:**
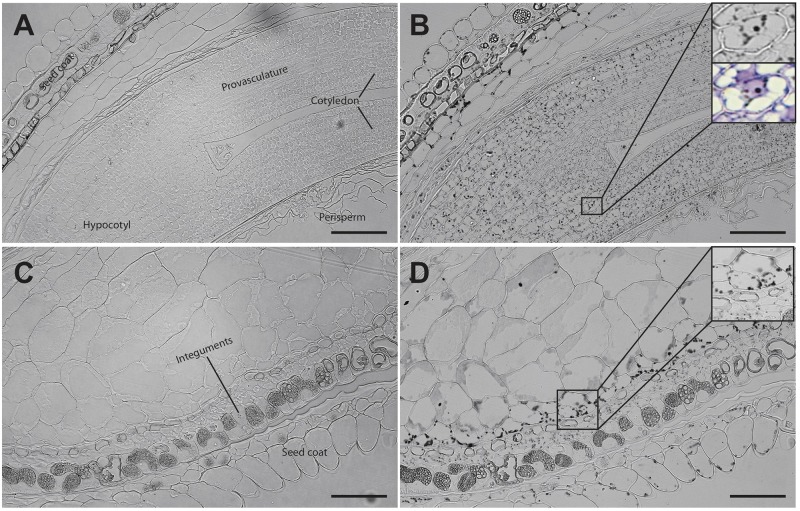
Iron distribution in *C. quinoa* seeds at cotyledon stage. *C. quinoa* seeds at the cotyledon stage (14 days postanthesis) dissected from fruits were embedded in Technovit resin, sectioned (3 μm) and then stained with Perls/DAB **(B**,**D)**. In **B** the zoom shows iron accumulation into and around the nuclei. In **B** and **D** toluidine blue was used to show different cell structures. Unstained sections were used as control **(A**,**C)**. The scale bar represents 100 μm.

Analysis of seed sections containing early embryo developmental stages revealed that there are no detectable iron pools in seed coat, perisperm or embryos (Figure 4). In seeds containing embryos at the cotyledon stage iron is detected inside the nuclei and in structures surrounding the nuclei (Figures 5A,B). Interestingly, longitudinal sections of the entire seed showed detectable iron pools in the integuments. Strong iron staining was observed in cytoplasmic structures (Figures 5C,D). In order to confirm the subcellular compartments that accumulate iron we performed Perls/DAB and Toluidine blue staining on the same sections. The zoom of specific cells in Figures 5B,D, show that iron localizes in nuclei and cytoplasmic structures in the embryo. Moreover, there is strong iron staining in cytoplasmic structures in the integuments.

**FIGURE 6:**
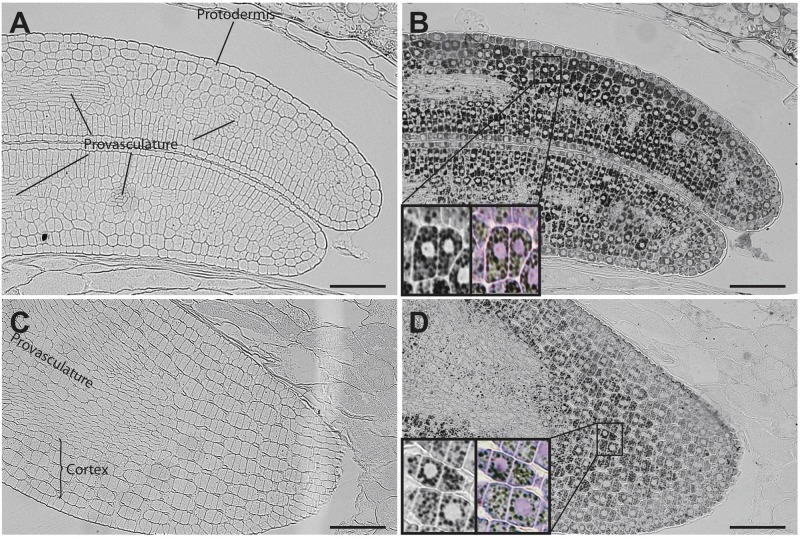
Iron distribution in *C. quinoa* seeds at mature embryo stage. *C. quinoa* seeds at the mature embryo stage, before seed desiccation stage (21 days postanthesis), were collected from fruits, embedded in Technovit resin and sectioned (3 μm). The sections were then stained with Perls/DAB **(B**,**D)**. Cotyledons and hypocotyl are shown in **A**,**B** and **C**,**D**, respectively. The zoom in **B** and **D** show iron accumulation in cell structures different from nuclei. Perls/DAB and Toluidine blue were used in order to reveal different cell structures. Unstained sections were used as control **(A**,**C)**. The scale bar represents 100 μm.

In mature seeds, iron is no longer localized in nuclei (Figure 6). At this stage, iron is detected in several embryonic cell types, including endodermis and cortex cells of cotyledons and hypocotyl (Figures 6B,D). Perls/DAB and Toluidine blue staining show that iron pools do not localize in nuclei in cotyledon and hypocotyl cells (Zoom in Figures 6B,D).

## Discussion

Dietary iron deficiency is a major issue for human health, affecting more than 2 billion people around the world (Murgia et al., 2012). Seeds are a pivotal source of iron for humans and animals (Roschzttardtz et al., 2017). Therefore, understanding the molecular mechanisms involved in iron loading and distribution in seeds is critical for the development of biotechnological approaches to improve seed iron content. Generation of biofortified crops could be a financially sound strategy to deliver nutrients to the population, as an alternative to the continuous governmental investment in fortification and supplementation programs (DeFries et al., 2015). Thus, genetic biofortification of staple crops could result in an environmentally friendly and cost-effective strategy for the improvement of nutritional health (Guerinot and Yi, 1994).

Pioneer studies, using SμXRF and a histochemical method for iron detection in plant tissues (Perls/DAB stain), showed that iron accumulates in the vacuoles of the endodermis cell layer of *A. thaliana* mature embryos (Kim et al., 2006; Roschzttardtz et al., 2009; Eroglu et al., 2017; Ibeas et al., 2017). While this distribution pattern is conserved in the rest of the Brassicaceae family (Ibeas et al., 2017), it is not present in legumes (Cvitanich et al., 2010). These observations suggest that embryos may have multiple ways to store iron. Consequently, by exploring this diversity, we should be able to propose suitable models to dissect the molecular mechanisms of seed iron storage and eventually translate these findings to crops of commercial and nutritional value. Toward this end, we used Perls/DAB staining to determine iron distribution in several plant species along the Eudicotyledoneae phylogeny. As a result, we obtained the first phylogenetic study of iron distribution in plant seed embryos. The results indicate that seed embryos belonging to different Eudicotyledoneae orders accumulate iron in several cell layers, with more diversity that what can be inferred by simply using Arabidopsis as model. In particular, cortex cells accumulate iron in all the embryo species analyzed except for Brassicales families Capparaceae, Cleomaceae and Brassicaceae. These results strongly suggest that iron distribution in cortex cells in embryos from Eudicotyledoneae embryo plants is a conserved characteristic from a phylogenetic point of view. We used S-μXRF as a second and independent method to confirm that in quinoa embryos, iron accumulates in several cell layers including the cortex and endodermis cells (Figure 3B).

Quinoa seed has high nutritional value and has recently started to be used as a novel functional food (Abugoch, 2009). This plant contains 15 mg of iron per 100 g of seeds, covering the daily iron needs of infants and adults (National Academy Press, 2001; Konishi et al., 2004). We evaluated iron accumulation during *C. quinoa* seed development and found that it does not accumulate iron in any seed tissue during the early developmental stages (Figure 4). This is in contrast with what was previously described for *B. napus* seeds, in which strong iron staining was observed in the nuclei of the free cell endosperm surrounding the embryo, and also in several cell layers of the embryo at the torpedo stage (Ibeas et al., 2017). This discovery opens new questions on the mechanisms of iron distribution and accumulation in these seeds, and will be used as the basis for the development of biotechnological strategies to increase total iron content in seeds for human consumption.

A side finding of this work is the broad distribution of Mn in the embryo of *C. quinoa* hypocotyl (Figure 3B), compared to the restricted location of Mn in the hypocotyl subepidermal tissue of *A. thaliana* embryos (Kim et al., 2006; Punshon et al., 2012; Schnell Ramos et al., 2013; Eroglu et al., 2017). It has recently been shown that mutations in the genes *cax1cax3* (Punshon et al., 2012), *mtp8* and *vit1* (Eroglu et al., 2017) cause a broad distribution of Mn indicating that they are responsible for this localization. As proposed for iron, this finding will open new strategies for the development of Mn fortified seeds.

In addition to Brassicales, our analyses also showed that at least some Zygophyllales, such as *P. chilensis*, also have an unusual iron distribution. In this case, iron is distributed inside and outside the cells (apoplast). This iron pool would necessarily be accumulated in a different manner from the vacuolar-located. For instance, there would be no need for particular metal transporters as is the case for Arabidopsis (Lanquar et al., 2005; Kim et al., 2006). It would also be interesting to determine the iron ligands, and how assimilable it is. Further analyses of this and other Zygophyllales species will be carried out to ascertain the extension of this phenotype and to determine its molecular bases.

**FIGURE 7:**
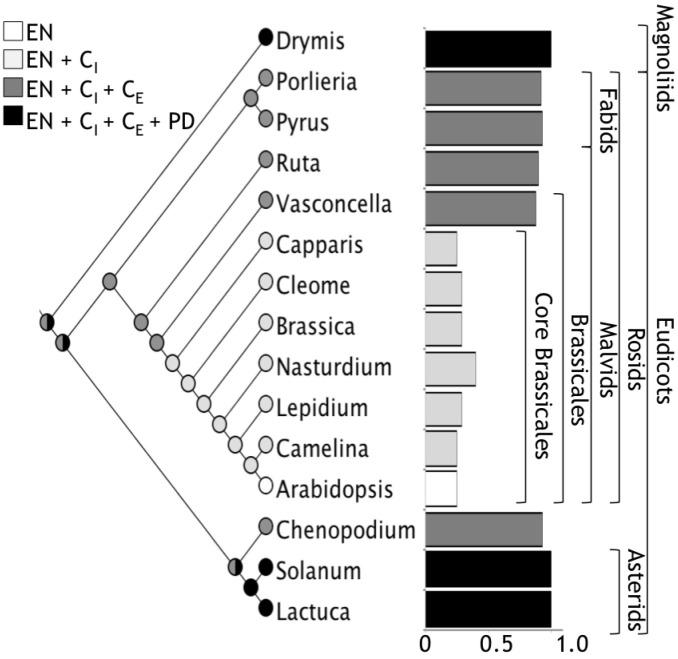
A global view of iron distribution in embryos. Phylogenetic tree showing all the species analyzed in this study. Colors of circles indicate type of cells that accumulate iron in the hypocotyl. White, only endodermis; light gray, endodermis + internal cortex (C_I_); dark gray, endodermis + internal cortex + external cortex (C_E_); black, endodermis + internal cortex + external cortex + protodermis (PD). Cortex cell are defined as the cell layers that are between the endodermis and the protodermis, we refer as internal cortex the cell layers than are near to the endodermis. Ratio of number of cells where iron is accumulated versus total number of cell layers from the endodermis to the protodermis for embryos from different orders are indicated in bars. Colors of bars correspond to the same code described above.

Regarding the widespread use of Arabidopsis as a Eudicotyledoneae model plant for iron nutrition, our results show that iron distribution in Arabidopsis embryos is not a widely conserved character in Eudicotyledoneae seed embryos and it corresponds to an apomorphic character. Ancestral reconstruction of the phylogeny of angiosperms with Maximum Parsimony or Maximum Likelihood indicated that the embryo of the ancestor of Brassicales, and probably of all Eudicotyledoneae, accumulates iron in several layers of the cortex (Figure 7). The loss of iron in the outer layers of the cortex evolved in the core of Brassicales, whereas the ability to accumulate iron only in endodermis is a derived trait of Arabidopsis. According to Beilstein et al. (2010), the apomorphy described in our study emerged between 120 and 70 Mya. In our opinion, Brassicales seeds could be used to evaluate molecular mechanisms involved in iron accumulation in the cortex cells, using information obtained from other species described in this article.

These results also open new questions on the mechanisms involved in iron accumulation and remobilization in cortex and protodermis cells, during embryogenesis and seed germination, respectively. From an evolutionary point of view, it will be interesting to study which competitive advantage determined the selection of this iron distribution in Brassicales species.

## Author Contributions

MI, SG-G, JV-P, NN, and HR performed the embryo dissections and Perls/DAB staining. IA, HC-M, and MG-G performed the SμXRF analysis. FP performed the phylogenetic analysis. All authors participated in the writing of the manuscript.

## Conflict of Interest Statement

The authors declare that the research was conducted in the absence of any commercial or financial relationships that could be construed as a potential conflict of interest.
