# Supplementary material for: The Diverse Iron Distribution in Eudicotyledoneae Seeds: From Arabidopsis to Quinoa
Source: Front Plant Sci. 2019 Jan 15;9:1985. doi: 10.3389/fpls.2018.01985 (PMC6341002; doi:10.3389/fpls.2018.01985)
Supplement: Supplementary file 1 [file Data_Sheet_1.PDF]

Supplemental table 1. List of seed embryos used in this study indicating the Order, Family and the name for each Species.

Order	Family	Species	Endodermis[a]	Cortex[a]	Protodermis[a]
**Brassicales**	Brassicaceae	*Arabidopsis thaliana*	X		
	Brassicaceae	*Camelina sativa*	X	X	
	Brassicaceae	*Nasturdium officinale*	X	X	
	Brassicaceae	*Lepidium sativum*	X	X	
	Brassicaceae	*Brassica napus*	X	X	
	Cleomaceae	*Cleome hassleriana*	X	X	
	Capparaceae	*Capparis spinosa*	X	X	
	Caricaceae	*Vasconcellea pubescens*	X	X	
**Sapindales**	Rutaceae	*Ruta graveolens*	X	X	
**Rosales**	Rosaceae	*Pyrus communis*	X	X	
**Zygophyllales**	Porlieria	*Porlieria chilensis*	X	X	
**Solanales**	Solanaceae	*Capsicum annuum*	X	X	
	Solanaceae	*Solanum lycopersicum*	X	X	X
**Asterales**	Asteraceae	*Lactuca sativa*	X	X	X
**Caryophyllales**	Polygonaceae	*Rumex acetosa*	X	X	X
	Polygonaceae	*Rheum rhaponticum*	X	X	
	Polygonaceae	*Fagopyrum esculentum*	X	X	
	Phytolaccaceae	*Phytolacca dioica*	X	X	
	Amaranthaceae	*Chenopodium quinoa*	X	X	
	Amaranthaceae	*Spinacia oleracea*	X	X	
	Amaranthaceae	*Beta vulgaris*	X	X	
**Canellales**	Winteraceae	*Drimys winteri*	X	X	X

[a]Letter X indicates detectable iron by Perls/DAB staining for each cell layer.
