# Supplementary figures and images for: The Diverse Iron Distribution in Eudicotyledoneae Seeds: From Arabidopsis to Quinoa
Source: Front Plant Sci. 2019 Jan 15;9:1985. doi: 10.3389/fpls.2018.01985 (PMC6341002; doi:10.3389/fpls.2018.01985)

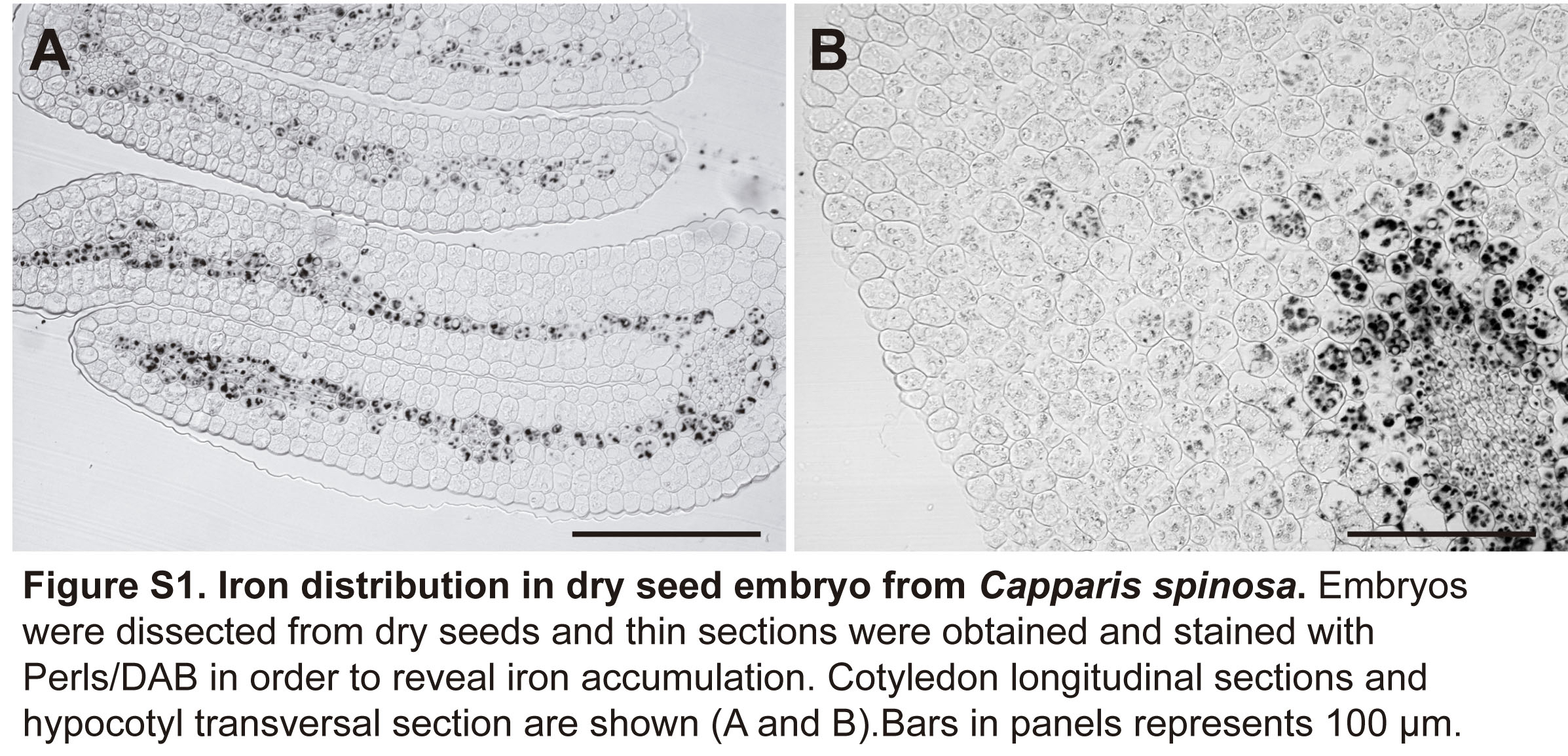

Supplement: Supplementary file 2 [file Image_1.JPEG]
